# Light-Induced Activation of a Specific Type-5 Metabotropic Glutamate Receptor Antagonist in the Ventrobasal Thalamus Causes Analgesia in a Mouse Model of Breakthrough Cancer Pain

**DOI:** 10.3390/ijms23148018

**Published:** 2022-07-20

**Authors:** Serena Notartomaso, Nico Antenucci, Francesca Liberatore, Giada Mascio, Stefano Vito Boccadamo Pompili, Joan Font, Mariarosaria Scioli, Livio Luongo, Antonietta Arcella, Roberto Gradini, Amadeu Llebaria, Ferdinando Nicoletti

**Affiliations:** 1 IRCCS Neuromed, 86077 Pozzilli, Italy; serena.notartomaso@neuromed.it (S.N.); francesca_liberatore@hotmail.it (F.L.); giada.mascio@neuromed.it (G.M.); mariarosariascioli@hotmail.it (M.S.); arcella@neuromed.it (A.A.); 2 Department of Physiology and Pharmacology, Sapienza University, 00185 Rome, Italy; nicoantenucci@gmail.com (N.A.); stefanovito.boccadamopompili@uniroma1.it (S.V.B.P.); 3 MCS, Laboratory of Medicinal Chemistry, Institute for Advanced Chemistry of Catalonia (IQAC-CSIC), 08034 Barcelona, Spain; joan.font@splice.bio (J.F.); amadeu.llebaria@iqac.csic.es (A.L.); 4 Department of Experimental Medicine, Division of Pharmacology, University of Campania “L. Vanvitelli”, 80138 Naples, Italy; livio.luongo@gmail.com; 5 Department of Experimental Medicine, Sapienza University, 00185 Rome, Italy; roberto.gradini@uniroma1.it

**Keywords:** breakthrough cancer pain (BTcP), optopharmacology, metabotropic glutamate receptor 5, analgesia, thalamus

## Abstract

Breakthrough cancer pain (BTcP) refers to a sudden and transient exacerbation of pain, which develops in patients treated with opioid analgesics. Fast-onset analgesia is required for the treatment of BTcP. Light-activated drugs offer a novel potential strategy for the rapid control of pain without the typical adverse effects of systemic analgesic drugs. mGlu5 metabotropic glutamate receptor antagonists display potent analgesic activity, and light-induced activation of one of these compounds (JF-NP-26) in the thalamus was found to induce analgesia in models of inflammatory and neuropathic pain. We used an established mouse model of BTcP based on the injection of cancer cells into the femur, followed, 16 days later, by systemic administration of morphine. BTcP was induced by injection of endothelin-1 (ET-1) into the tumor, 20 min after morphine administration. Mice were implanted with optic fibers delivering light in the visible spectrum (405 nm) in the thalamus or prelimbic cortex to locally activate systemically injected JF-NP-26. Light delivery in the thalamus caused rapid and substantial analgesia, and this effect was specific because light delivery in the prelimbic cortex did not relieve BTcP. This finding lays the groundwork for the use of optopharmacology in the treatment of BTcP.

## 1. Introduction

Breakthrough cancer pain (BTcP) refers to a sudden and transient exacerbation of pain, which occurs on a background of adequately controlled pain [1]. BTcP has a strong impact on healthcare and quality of life [2,3,4] and is difficult to treat. Fast-onset analgesic drugs (e.g., transmucosal fentanyl or intravenous opioids) are effective in the treatment of BTcP, although there is a conceptual paradox because patients with cancer who develop BTcP are often under chronic treatment with opioids. An attractive possibility is that patients might push a button at the onset of BTcP and deliver light into a selected region of the pain neuraxis to locally activate a circulating inactive analgesic prodrug with high spatiotemporal resolution. This will be translated into immediate relief of pain with no systemic adverse effects. The pitfall of this strategy is the neurosurgical procedure required for the implantation of optic fibers in the CNS, but this may not be an “impassable barrier” for cancer patients with a limited life expectancy, considering that a substantial proportion of cancer decedents are not prescribed breakthrough medication during palliative care [5]. Here, optopharmacology may provide a means of intervening in BTcP.

Application of optopharmacology to the control of pain is an emerging field in neuroscience, and light-sensitive ligands of metabotropic glutamate (mGlu) receptors have been tested in several preclinical models of chronic pain [6,7]. mGlu receptors, which are glutamate receptors coupled to G proteins, form a family of eight subtypes, subdivided into three groups based on their amino acid sequence, transduction mechanisms, and pharmacological profile of activation/inhibition. Group-I mGlu receptors (mGlu1 and mGlu5) are coupled to G_q/11_ proteins, whereas group-II (mGlu2 and mGlu3), and group-III (mGlu4, mGlu6, mGlu7, and mGlu8) mGlu receptors are coupled to G_i/o_ proteins [8]. Most mGlu receptor subtypes play a key role in the regulation of pain transmission and are candidate drug targets for the treatment of chronic pain [9,10,11,12,13,14]. mGlu5 receptors have been extensively studied at different points in the pain neuraxis, where they are involved in mechanisms underlying nociceptive sensitization [9,13]. Negative allosteric modulators (NAMs) of mGlu5 receptors have consistently shown analgesic activity in animal models of inflammatory or neuropathic pain [10,14], and the mGlu5 NAM, fenobam, was shown to reduce nociceptive sensitization in humans [15]. Another mGlu5 NAM, raseglurant, showed therapeutic efficacy against migraneous pain in a placebo-controlled clinical trial [16].

We developed an inactive photocaged derivative of raseglurant, compound JF-NP-26, which is converted into raseglurant by violet light illumination. Using this compound, we could demonstrate that optical control of mGlu5 receptors may provide a new strategy for the experimental treatment of chronic pain. For example, in mice developing neuropathic pain in response to sciatic nerve ligation and systemically injected with JF-NP-26, bilateral delivery of light in the visible spectrum (VS, 405 nm) in the ventrobasal thalamus caused prompt and substantial analgesia, which was even greater than that produced by systemic administration of raseglurant [6]. The use of a compound activated by VS light is particularly valuable from a translational standpoint because, as opposed to UV light, VS light dose not damage brain tissue. Here, we examined whether light-activated JF-NP-26, in two regions of the pain neuraxis (the ventrobasal thalamus and the prelimbic cortex), could induce rapid analgesia in an established animal model of BTcP.

## 2. Results

**Figure 1:**
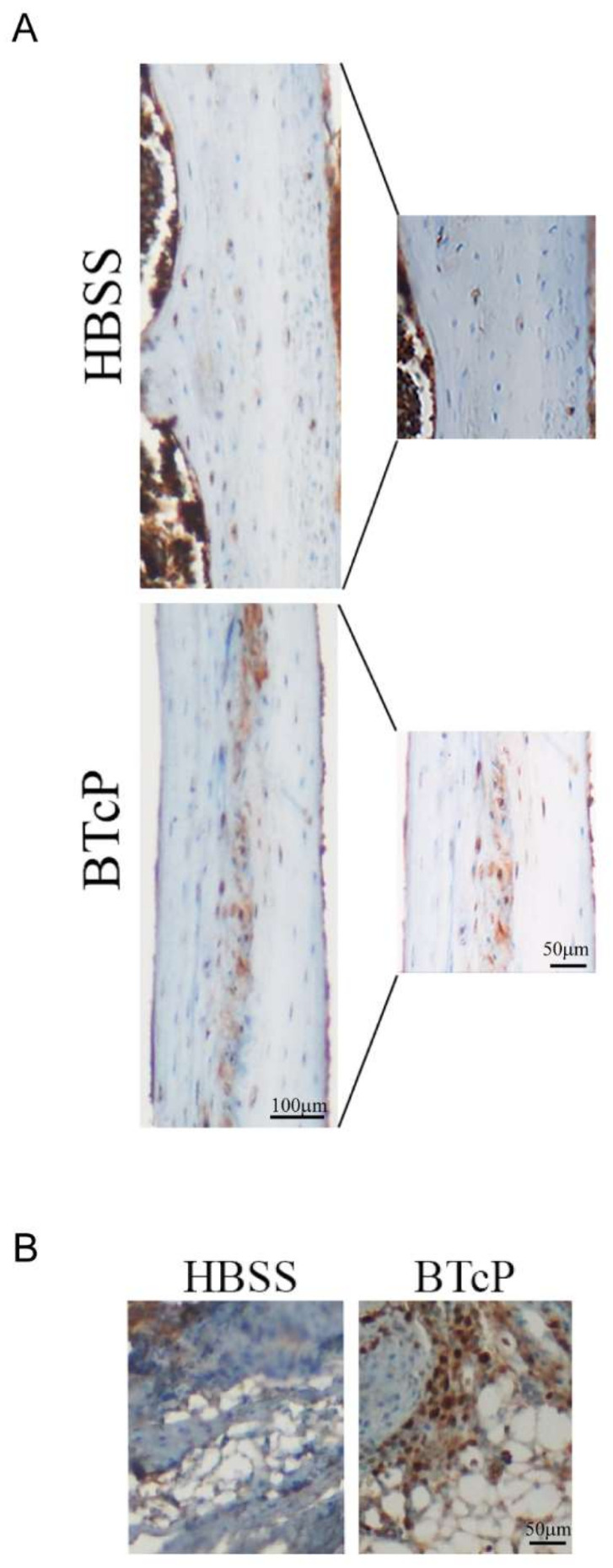
Proliferating cancer lung cells in the femur of BTcP mice. Cytokeratine immunostaining in representative tissue sections from the femur of control mice locally injected with HBSS and mice injected with LLC cells (BTcP mice) is shown in (**A**). Ki-67 immunostaining is shown in (**B**).

Figure 1 shows the presence of proliferating lung cancer cells in the femur of mice locally implanted with LLC-1 cells, as detected by the presence of the epithelial cell marker, cytokeratine (Figure 1A), and the cell proliferation marker, Ki-67 (Figure 1B).

**Figure 2:**
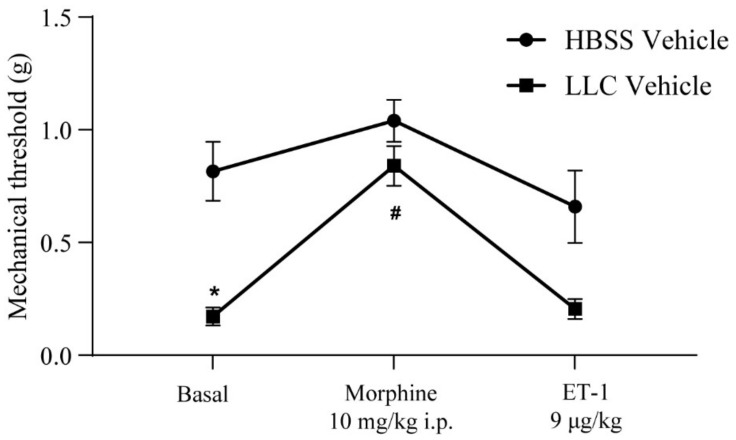
Assessment of mechanical pain thresholds in BTcP and control mice. Paw withdrawal thresholds in BTcP and control (HBSS) mice were recorded 10 min before i.p. injection of morphine (10 mg/kg), 20 min after morphine injection, and 5 min after intrafemoral injection of ET-1 (9 µg/kg). Data are means ± SEMs of 10 mice per group. Two-way ANOVA for repeated measures: HBSS vs. BTcP, F (1, 18) = 12.10; *p* = 0.0027; treatment, F (1.462, 26.31) = 37.51; *p* < 0.0001; interaction, F (2, 36) = 6.059; *p* = 0.0054. Sidak’s post hoc test *p* < 0.05 vs. the corresponding value of the HBSS group (*) or vs. basal and ET-1 value of the same group (#).

Mice unilaterally implanted with lung carcinoma cells in the femur showed a substantial reduction in mechanical pain thresholds after 16 days compared to control mice receiving HBSS injection in the femur (Figure 2). This model of cancer pain was highly sensitive to opioids, as shown by the substantial analgesic effect caused by a single i.p. injection of morphine (10 mg/kg) (Figure 2). Local injection of endothelin-1 (ET-1, 9 μg/kg) in the site of tumor growth, 20 min after morphine injection, completely abolished morphine-induced analgesia, with mechanical thresholds returning to pre-morphine values after 5 min (Figure 2). ET-1 injection had no significant effect on pain thresholds in control mice receiving HBSS in the femur (Figure 2).

**Figure 3:**
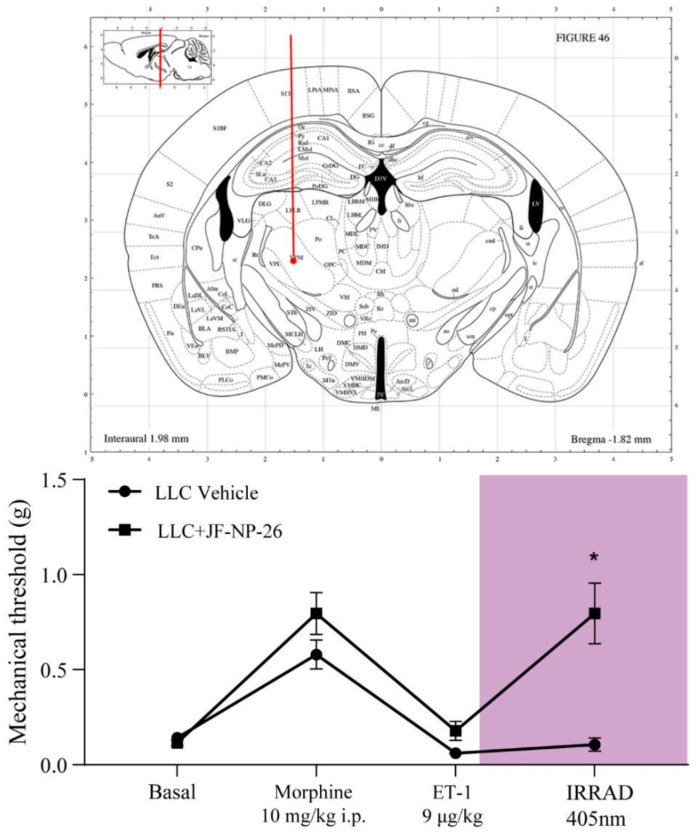
Light-induced activation of JF-NP-26 in the ventrobasal thalamus causes rapid analgesia in BTcP mice. Paw withdrawal thresholds were recorded 10 min before i.p. injection of morphine (10 mg/kg), 20 min after morphine injection, 5 min after intrafemoral injection of ET-1 (9 µg/kg), and 5 min after bilateral light delivery (“IRRAD”). Values are means ± S.E.M. of 10 mice per group. Two-way ANOVA for repeated measures: Vehicle vs. JF-NP-26, F (1, 18) = 11.91; *p* = 0.0028; treatment, F (2.031, 36.55) = 34.11; *p* < 0.0001; interaction, F (3, 54) = 10.88; * *p* < 0.0001. Sidak’s post hoc test, *p* < 0.05 vs. the corresponding value of vehicle treated mice.

To examine the efficacy of light-induced activation of JF-NP-26 in the ET-1 BTcP model, different groups of mice were bilaterally implanted with optic fibers in the ventral basal thalamus or in the prelimbic cortex, two brain regions in which mGlu5 receptors are involved in pain transmission and nociceptive sensibilization [17,18]. Light was delivered at a wavelength of 405 nm, which has been identified as the optimal VS wavelength causing photolysis of JF-NP-26, into the active mGlu5 receptor NAM, raseglurant [6]. Light delivery into the thalamus 5 min following ET-1, caused fast analgesia in mice that had been treated systemically with JF-NP-26 (10 mg/kg, i.p., injected at the same time as morphine) (Figure 3). Intrathalamic irradiation did not change pain thresholds in mice that were injected systemically with vehicle instead of JF-NP-26 (Figure 3).

**Figure 4:**
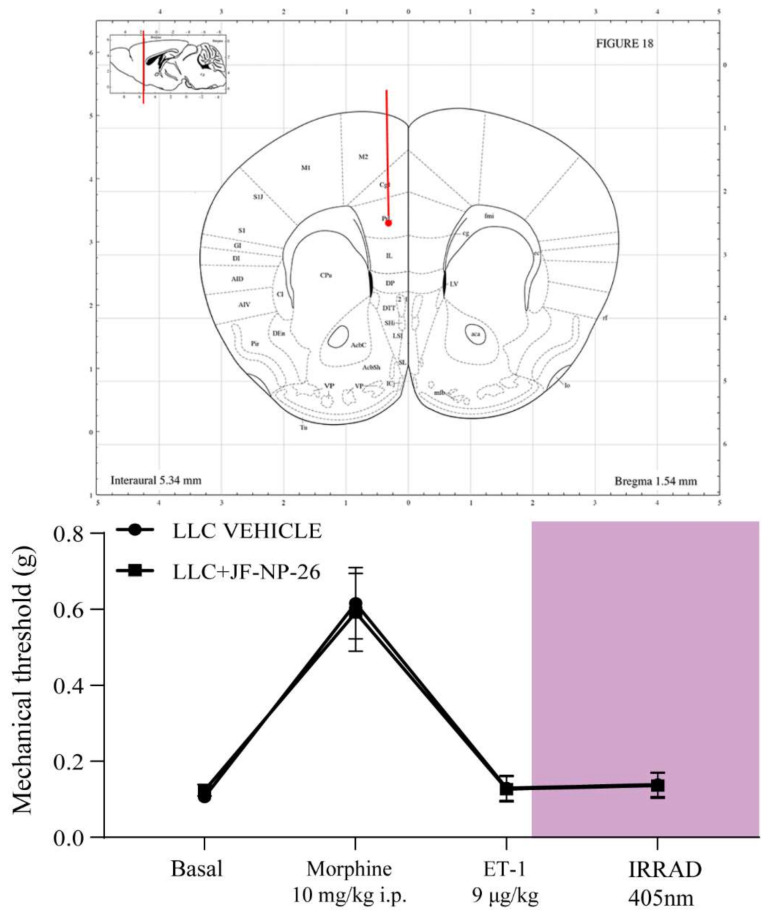
Light-induced activation of JF-NP-26 in the prelimbic cortex failed to induce analgesia in BTcP mice. Mice were treated as in Figure 3, with the difference that irradiation was performed bilaterally in the prelimbic cortex. Values are means ± S.E.M. of 10 mice per group.

Interestingly, bilateral light delivery in the prelimbic cortex of JF-NP-26-injected mice failed to cause analgesia (Figure 4), suggesting that mGlu5 receptors in the medial prefrontal cortex have a limited role in the pathophysiology of breakthrough pain.

## 3. Discussion

The present findings raise the attractive possibility that optopharmacology might be applied to the treatment of breakthrough pain. The use of light-activated drugs may ensure a highly localized effect of analgesic agents resulting in an optimal risk-to-benefit ratio in the treatment of breakthrough pain. As highlighted in the Introduction, mGlu5 receptors are considered as candidate drug targets for the treatment of pain [13,14,15,16], with mGlu5 receptor NAMs showing consistent analgesic activity in models of inflammatory or neuropathic pain [19]. The use of these drugs, however, is limited by their potential impact on synaptic transmission and mechanisms of activity-dependent synaptic plasticity underlying learning and memory processes [15,19]. These potential pitfalls are mitigated by local activation of mGlu5 receptor NAMs in the thalamus, which is the main relay station of the ascending pain pathway but has a limited role in cognitive functions. We were surprised to find that local activation of JF-NP-26 in the prelimbic cortex, which, in mice, is part of the medial prefrontal cortex [20], failed to cause analgesia. The role of mGlu5 receptors in pain control has been extensively investigated in the prelimbic cortex and other regions of the medial prefrontal cortex (i.e., the anterior cingulate cortex and the infralimbic cortex), and it is currently believed that mGlu5 receptor blockade in these regions causes analgesia [17]. Our data suggest that mGlu5 receptors in the prelimbic cortex are not critically involved in the pathophysiology of breakthrough pain. Perhaps, the treatment of breakthrough pain, which is an extremely severe form of pain, requires pharmacological modulation of a brain region that has a non-redundant role in pain control, such as the thalamus.

**Figure 5:**
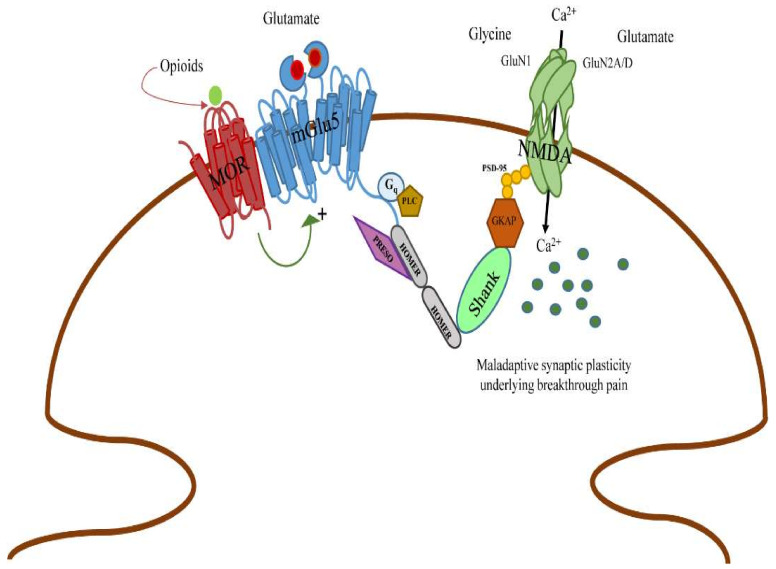
Hypothetical menage-a-trois among MOR, mGlu5 and NMDA receptors in the pathophysiology of breakthrough pain. MOR opioid receptors form functional heterodimers with mGlu5 receptors [21]. mGlu5 receptors, in turn, are physically linked to NMDA receptors via scaffolding proteins (long isoforms of Homer, Shank, GKAP, and PSD-95). Activation of mGlu5 receptors is known to facilitate NMDA receptor activation through protein kinase C and other mechanisms [23]. We hypothesize that opioid treatment supports the activity of NMDA receptors (via mGlu5 receptors), leading to enhanced calcium influx and activation of calcium-dependent enzymes (not shown). This may result in a maladaptive form of synaptic plasticity in nociceptive thalamic neurons underlying breakthrough pain.

An attractive hypothesis is that thalamic mGlu5 receptors mediate the expression of a maladaptive form of synaptic plasticity that is induced by chronic activation of opioid receptors within the context of cancer pain. Interestingly, recent findings indicate that MOR opioid receptors and mGlu5 receptors form functional heterodimers that can be targeted by analgesic drugs in bone cancer pain [21]. NMDA receptors are involved in the pathophysiology of breakthrough pain which develops during treatment with opioids [22], and mGlu5 receptors are physically and functionally linked to NMDA receptors [23]. Opioid, mGlu5, and NMDA receptors may form *a mènage-a-trois*, which underlies the development of breakthrough pain (Figure 5); selective pharmacological blockade of mGlu5 receptors in the thalamus may disrupt the link among these receptors, producing fast and substantial analgesia.

## 4. Materials and Methods

### 4.1. Reagents

Morphine hydrochloride injection solution (10 mg/mL) was purchased from Farmacie Internazionali di Parisi Fernanda e Ninni Barbara Sas (Napoli, Italy). ET-1 was purchased from Sigma-Aldrich (St. Louis, MO, USA) and dissolved in distilled deionized water (DD water) and injected at a dose of 9 μg/kg, (75 pmol/10 μL). Dulbecco’s modified Eagle’s medium (DMEM) with high glucose, D-Hank’s balanced salt solution (HBSS) and fetal bovine serum were purchased from Gibco Company (Gaithersburg, MD, USA). JF-NP-26, (10 mg/kg, i.p., dissolved in saline containing 6% DMSO and 6% Tween-80) was synthesized by A.L. An in-depth characterization of JF-NP-26 is reported in [6].

### 4.2. Animals

We used adult (8–12 week) male C57Bl/6 mice. Mice were housed (2–5 per cage) on a standard 12-h light-dark cycle (lights on at 6:00 a.m.) under controlled conditions (temperature, 22 °C; humidity, 40%) with food and water ad libitum. Studies were performed in accordance with national and international guidelines and regulations on animal care and use and were approved by the Neuromed Institutional Animal Care and Use Committee and by the Italian Ministry of Health (804/2018-PR).

### 4.3. Cell Culture

LL/2 (LLC1) Lewis lung carcinoma cells (a murine lung adenocarcinoma cell line) were obtained from ATCC (#crl-1642 Manassas, VA, USA). Cells were cultured with DMEM (high glucose) supplemented with 10% FBS in a humidified incubator with 5% CO_2_ at 37 °C and grown to ~80% confluence in 75 cm^2^ flasks with medium changes every 2 days, as described previously [24]. Then, cells were trypsinized, centrifuged at 600× *g*, and suspended at a final concentration of 2 × 10^6^/μL in D-Hank’s solution prior to implantation in the mouse femur.

### 4.4. Brain Optic Fiber Implantation

Mice were anesthetized with isoflurane (5% for induction and 2% for maintenance) and implanted with optic fibers (TFC_400/430–0.53_3.5 mm_TSM4.0_B45; TFC_400/430–0.53_2.7 mm_TSM4.0_B45, Doric Lenses Inc., Quebec, Canada) using dental cement and surgical screws (Agnthos, Lidingö, Sweden) in a Stoelting Kopf stereotaxic frame (Stoelting Co., Wood Dale, IL, USA). The sites of implantation were both the left and right thalamus (coordinates: −1.8 mm posterior to the bregma, ±1.5 mm lateral to the midline, 3.5 mm ventral from the surface of skull) or the prelimbic cortex (coordinates: +1.50 mm posterior to the bregma, ±0.3 mm lateral to the midline, 2.7 mm ventral from the surface of skull) according to the atlas of Paxinos and Franklin [25]. Body temperature was monitored and maintained at 37 °C with a rectal thermistor coupled to a heating blanket. Animals were kept for 2 h at 37 °C before being transferred to their home cages. For sham operation, animals were subjected to the same anesthesia and surgical procedures.

### 4.5. Mouse Model of BTcP

During the same anesthesia procedure, 10 μL (2 × 10^6^/μL) of LLC cell suspension was injected into the femur of the left hind limb with a microinjector for the induction of bone cancer pain as described previously [24]. Control mice were injected with HBSS with no cells in the femur. At post-implantation day 16, mice were treated systemically with morphine (10 mg/kg, i.p.) followed, 20 min later, by 20 μL of ET-1 (9 μg/kg, 75 pmol/10 μL), injected with a microsyringe into the same site of LLC implantation.

### 4.6. Assessment of Mechanical Pain Thresholds

Mechanical allodynia was assessed 16 days after surgery by measuring the hind paw withdrawal response to von Frey filament stimulation. Mice were placed in a dark box (20 × 20 × 40 cm) with a wire grid bottom through which the von Frey filaments (North Coast Medical, Inc., San Jose, CA, USA), bending force range from 0.008 to 3.5 g, were applied by using a modified version of the up-down paradigm described previously [26]. Each filament was applied and pressed perpendicularly to the plantar surface of the hind paw until it bent for five times with a 3 min interval. The filament that evoked at least three paw withdrawals was assigned as the pain threshold in grams.

### 4.7. Experimental Design and Drug Administration

The study was designed as follows: (i) LLC cells were injected in the femur and optic fibers were implanted in the ventrobasal thalamus or prelimbic cortex at the same time; (ii) after 16 days, mice were treated i.p. with morphine combined with the light-sensitive mGlu5 receptor NAM, JF-NP-26 (10 mg/kg), or its vehicle; (iii) after 20 min, ET-1 (9 μg/kg) was injected in the tumor mass for the induction of BTcP; and, (iv) 405 nm light (2000 mA, intensity, and 500 Hz, frequency) was delivered in the thalamus or prelimbic cortex 5 min after ET-1 injection (Supplementary Figure S1). Mechanical pain thresholds were detected 10 min prior to morphine injection, 20 min following morphine injection, 5 min following ET-1 injection (just prior to light delivery), and 5 min following light delivery. At the end of pain assessment, mice were euthanized, and the femurs were removed for immunohistochemical analysis.

### 4.8. Immunohistochemical Analysis

Femur and tibiae bones were fixed in 4% PBS-buffered paraformaldehyde overnight at 4 °C. The samples were then stored in 70% alcohol. For paraffin sections, samples were decalcified in 15% EDTA for 2 weeks and then dehydrated in alcohol, cleared with xylene, and embedded in paraffin. Five-μm-thick sections were cut using microtome (Leica Microsystems Nussle GmbH, Wetzlar, Germany).

Immunohistochemistry was carried out using antibodies directed against pan-cytokeratin (Roche, Basel, Switzerland dilution 1:100) and Ki-67 (Invitrogen; Waltham, Massachusetts, USA; dilution 1:100). Immunostaining was performed using standard protocols with a Leica Bond automated immunostainer, followed by antibody detection using a Leica Polymer Kit and diaminobenzidine as a chromogen. Stained slides were photographed under a light microscope (Olympus Microsystems, Tokyo, Japan).

### 4.9. Statistics

Statistical analysis was performed by two-way ANOVA for repeated measures and Sidak’s post hoc test. A value of *p* < 0.05 was considered as statistically significant.

## 5. Conclusions

Prodrugs activated by light in specific brain regions may offer a new strategy for the treatment of severe, sporadic, pain, which is difficult to manage with conventional analgesic agents. We have shown that one of these drugs, a caged derivative of the mGlu5 receptor NAM, raseglurant, was highly effective in reducing BTcP when locally activated in the ventrobasal thalamus. This finding lays the groundwork for future studies in which this approach may be compared with other established strategies for the treatment of BTcP in terms of efficacy, safety, and tolerability.

## Data Availability

Not applicable.

## Supplementary Materials

The following supporting information can be downloaded at: https://www.mdpi.com/article/10.3390/ijms23148018/s1.
