# Supplementary material for: Light-Induced Activation of a Specific Type-5 Metabotropic Glutamate Receptor Antagonist in the Ventrobasal Thalamus Causes Analgesia in a Mouse Model of Breakthrough Cancer Pain
Source: Int J Mol Sci. 2022 Jul 20;23(14):8018. doi: 10.3390/ijms23148018 (PMC9323585; doi:10.3390/ijms23148018)
Supplement: Supplementary file 1 [file ijms-23-08018-s001.zip › ijms-1816810-supplementary.pdf]

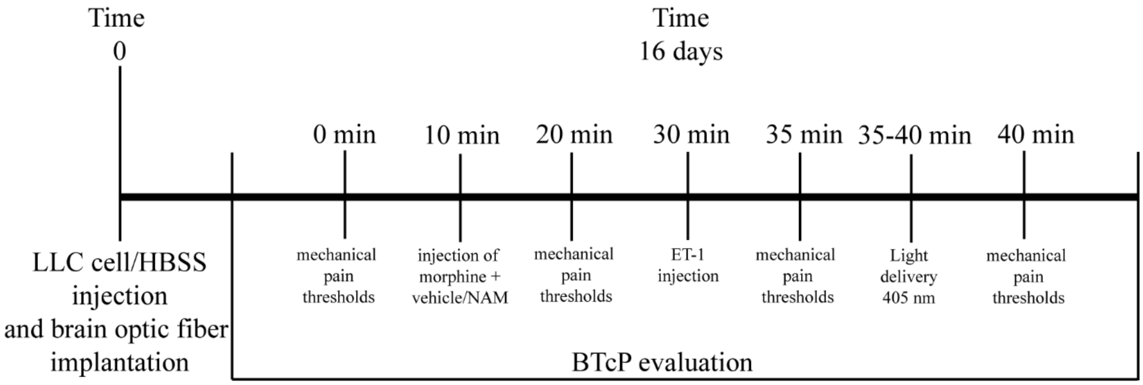

Figure S1 Time line of the experiment.

LLC (Lewis Lung Carcinoma cells); HBSS (D-Hank's balanced salt solution); BTcP (breakthrough cancer pain);
